# Twist-Angle-Dependent Electronic Properties of Exfoliated Single Layer MoS_2_ on Au(111)

**DOI:** 10.1021/acs.nanolett.3c02804

**Published:** 2023-10-16

**Authors:** Ishita Pushkarna, Árpád Pásztor, Christoph Renner

**Affiliations:** Department of Quantum Matter Physics, Université de Genève, 24 Quai Ernest Ansermet, CH-1211 Geneva, Switzerland

**Keywords:** twist angle, heterostructures, moiré, MoS_2_, Au(111), exfoliation, scanning tunneling microscopy, scanning tunneling spectroscopy

## Abstract

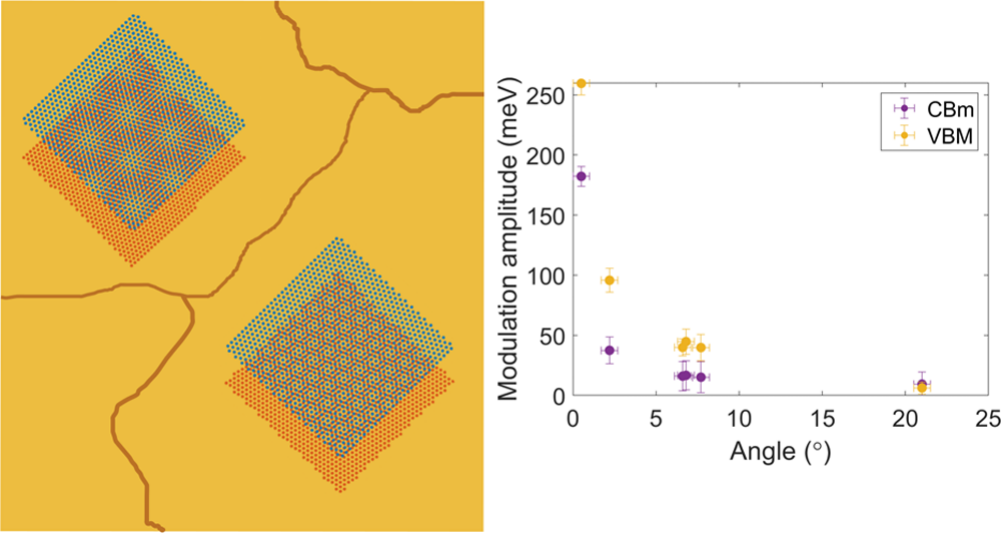

Synthetic materials and heterostructures obtained by the controlled stacking of exfoliated monolayers are emerging as attractive functional materials owing to their highly tunable properties. We present a detailed scanning tunneling microscopy and spectroscopy study of single layer MoS_2_-on-gold heterostructures as a function of the twist angle. We find that their electronic properties are determined by the hybridization of the constituent layers and are modulated at the moiré period. The hybridization depends on the layer alignment, and the modulation amplitude vanishes with increasing twist angle. We explain our observations in terms of a hybridization between the nearest sulfur and gold atoms, which becomes spatially more homogeneous and weaker as the moiré periodicity decreases with increasing twist angle, unveiling the possibility of tunable hybridization of electronic states via twist angle engineering.

The rapid increase in the number of 2D materials which can be exfoliated and the fantastic progress in their layer-by-layer stacking with controlled sequence^1^ and twist angle^2,3^ open up exceptional opportunities to design new functional quantum materials. A famous example is twisted bilayer graphene, whose very rich phase diagram ranges from correlated insulating to superconducting phases depending on twist angle and electrostatic doping.^4,5^ Heterostructures with unique properties can also be obtained by stacking 2D exfoliated transition metal dichalcogenides (TMD).^6−9^

Exfoliated 2D materials in direct proximity to a metal surface with selected twist angles offer further attractive materials engineering perspectives.^10^ Such structures are challenging to prepare with a clean surface suitable for scanning tunneling microscopy (STM). Therefore, many STM studies to date have been carried out on epitaxial thin films grown in situ by molecular beam epitaxy (MBE) or by chemical vapor deposition (CVD). The twist angle with the substrate of such films is set by thermodynamics and cannot be tuned at will. It is nearly 0° for 2H-MoS_2_ evaporated on Au(111),^11−13^ one of the most studied TMD on a metallic substrate.^14,15^

Manual stacking of exfoliated monolayers allows us to select arbitrary twist angles between the 2D material and the metallic substrate. Only a few STM studies of exfoliated 2H-MoS_2_ monolayers (hereafter simply MoS_2_) on Au(111) have been published,^16−22^ without any strong focus on twist-angle-dependent properties. Here, we present a detailed STM and scanning tunneling spectroscopy (STS) investigation of the electronic properties of MoS_2_ on Au(111) as a function of the twist angle. High-quality heterostructures allow us to explore genuine twist-angle-dependent properties. We find that the system is strongly hybridized independently of the twist angle and that the valence and conductance bands are modulated at the moiré period. The modulation amplitude vanishes with increasing twist angle, which we explain in terms of a hybridization between the sulfur and gold atoms that becomes spatially more homogeneous and weaker as the moiré periodicity decreases with increasing twist angle.

We performed STM and STS on continuous, millimeter-sized monolayer (ML) flakes obtained by exfoliating 2H-MoS_2_^23^ onto template-stripped Au substrates.^24^ These substrates are polycrystalline Au(111) films stripped from an ultraflat silicon wafer (see Methods). STM and X-ray diffraction measurements show that they consist of Au(111) oriented grains, where different grains can be slightly tilted and rotated about their [111]-axis (SI Section I). ML MoS_2_ on Au(111) is identified by its characteristic Raman spectrum^12,25,26^ and from optical images where MoS_2_ appears darker on a bright Au substrate^27^ (see SI Section I). A further confirmation that we have ML MoS_2_ is the observation of a moiré pattern in STM topography, which is absent for bilayer and thicker MoS_2_ flakes.^11^

Exfoliating large area MoS_2_ MLs onto polycrystalline Au(111) naturally produces regions with different twist angles between the two lattices. This provides a unique platform to characterize the electronic structure for different twist angles in a single sample, thus excluding potential sample-dependent fluctuations. Figure 1 shows typical raw data topographic STM images acquired in different regions of such a sample. They reveal the MoS_2_ lattice and the moiré patterns specific to the local twist angle (Figure 1a–d and SI Section II). The corresponding Bragg and moiré peaks are highlighted in the Fourier transform (FT) of the 7.7° heterostructure by green and red circles, respectively (Figure 1f). The complete data set of Figure 1 is consistent with a large single crystal MoS_2_ ML, with a lattice oriented along a single direction, including across step edges and across gold grain boundaries (Figure 1e and SI Section III). The slight distortion of the moiré pattern in Figure 1a is due to drift in this topographic image (see SI Section IV).

**Figure 1:**
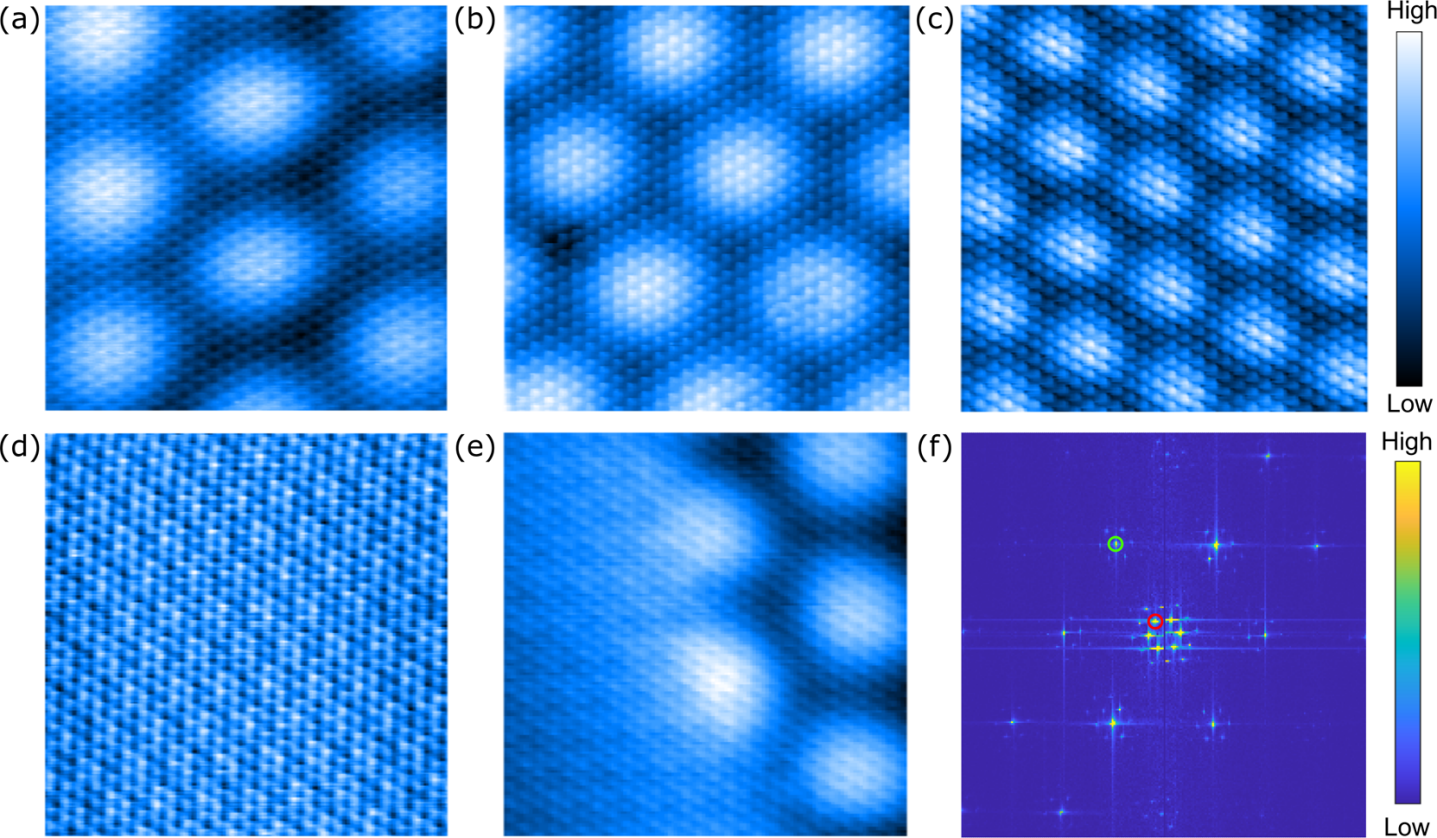
8 × 8 nm^2^ high resolution STM topography of MoS_2_ on Au(111) with a twist angle of (a) 0.5° (set point *I*_t_ = 200 pA; *V*_b_ = 500 mV), (b) 2.2° (500 pA; 100 mV), (c) 7.7° (200 pA; 300 mV) and (d) 21° (100 pA; 100 mV). (e) Domain boundary between a 21° and a 0.5° twist angle region (100 pA; 1 V). (f) FT of (c) with the MoS_2_ lattice and the moiré peaks identified with green and red circle, respectively.

We now turn to the spectroscopic characterization of MoS_2_ on Au(111) as a function of the twist angle. Typical *I*(*V*) spectra measured on our devices are shown in Figure 2a. They can be described as a modified semiconducting spectrum with a finite conductance in the gap region.^17,28^ We do not observe the spread in tunneling characteristics reported in previous STM experiments,^17^ most likely due to a cleaner MoS_2_/Au(111) interface exemplified by the perfect adherence of MoS_2_ to the substrate across grain boundaries and step edges (SI Section III). We do find occasional bubbles,^16,18^ where MoS_2_ is locally decoupled from the substrate (SI Section V). These regions are electronically different, with a fully gaped semiconducting *I*(*V*) spectrum (Figure 2a) consistent with previous STM studies where MoS_2_ was not in direct contact with a metal surface.^18,29−31^

**Figure 2:**
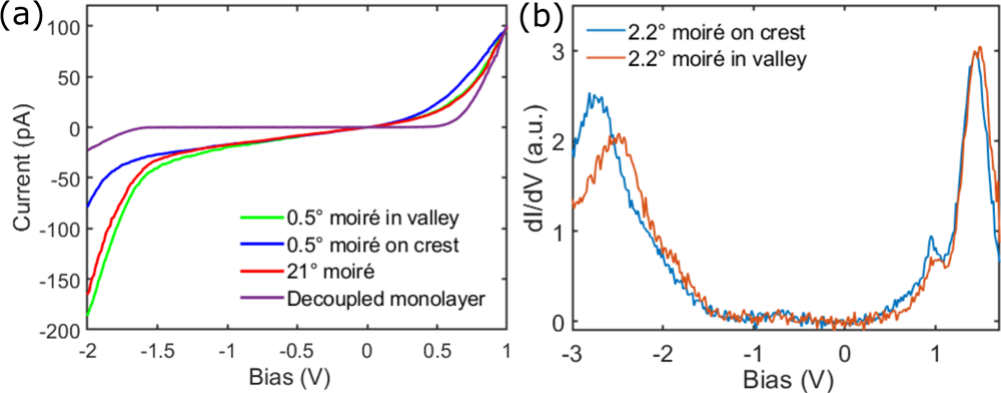
(a) *I*(*V*) tunneling spectra measured at a valley (green) and at a crest (blue) location of a 0.5° moiré pattern, on a 21° moiré pattern (red), and in a decoupled region of the MoS_2_ ML (purple). (b) d*I*/d*V*(*V*) spectra measured at a crest (blue) and at a valley (orange) location of a 2.2° twist angle moiré pattern.

While the generic line shape is the same for all *I*(*V*) tunneling spectra measured on our MoS_2_/Au(111) heterostructures, we find some variations, in particular as a function of twist angle and as a function of position in the moiré unit. They are most prominent at negative bias below the Fermi level (*E*_F_), which corresponds to *V*_Bias_ = 0 V in Figure 2. To characterize the twist angle dependence of the electronic properties of the MoS_2_/Au(111) heterostructures, we acquire *I*(*V*, *r⃗*) and d*I*/d*V*(*V*, *r⃗*) maps. For every tunneling conductance spectrum—two typical examples are shown in Figure 2b—, we fit the main peaks below (above) *E*_F_ to a Gaussian and define the valence band maximum VBM(*r⃗*) (conduction band minimum CBm(*r⃗*)) as the peak position plus (minus) its corresponding 2.2σ. Since the conductivity measured at these energies is predominantly related to states derived from MoS_2_,^32^ we define Δ(*r⃗*) = CBm(*r⃗*) – VBM(*r⃗*) as the gap. We find that Δ amounts to about 2 eV in agreement with previous findings for MoS_2_ MLs grown on Au(111).^33,34^ These three quantities are plotted alongside the corresponding topography for two different twist angles in Figure 3a–h. Note that the same information can be obtained from the *I*(*V*, *r⃗*) curves using a different fitting procedure (SI Section VI) used in Figure 3i–l and Figure 4b–d.

**Figure 3:**
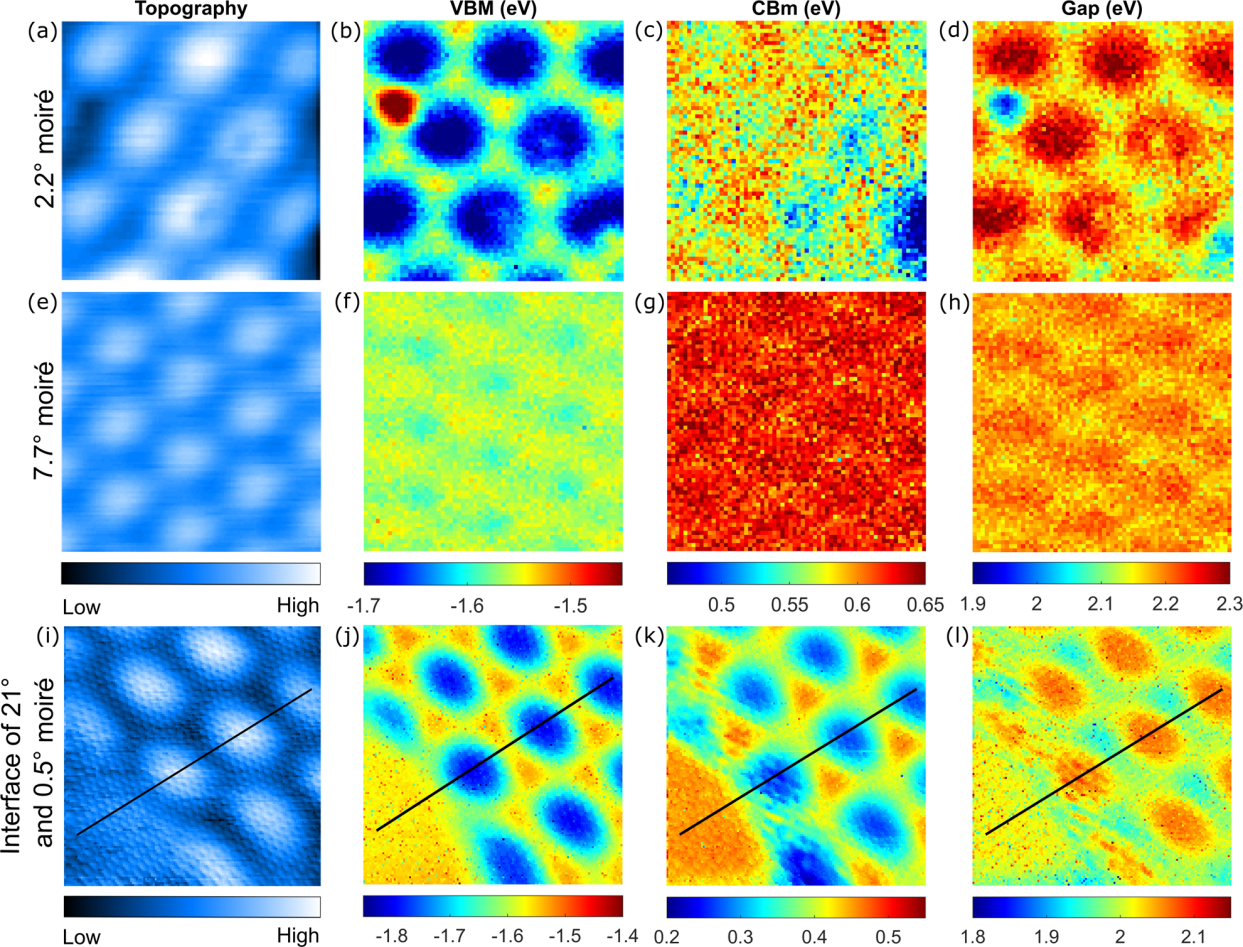
(a) 8 × 8 nm^2^ STM topography of a 2.2° twist angle heterostructure (set point: *I*_t_ = 100 pA, *V*_b_ = 1.7 V) and corresponding (b) VBM, (c) CBm, and (d) gap map. (e) 6 × 6 nm^2^ STM topography of a 7.7° twist angle heterostructure (100 pA, 1.0 V) and corresponding (f) VBM, (g) CBm, and (h) gap map—the color scales for both twist angles are at the bottom of the second row; set-point for the d*I*/d*V*(*V*, *r⃗*) maps was *I*_t_ = 100 pA and *V*_b_ = 1.7 V. (i) 9 × 9 nm^2^ STM topography of an interface between a 21° (bottom left corner) and a 0.5° twist angle heterostructure (100 pA, 1.0 V) and corresponding (j) VBM, (k) CBm, and (l) gap map (extracted from *I*(*V*, *r⃗*) spectra acquired with set point: 100 pA, 1.0 V). The distortion in panels i–l is due to drift during the long data acquisition time (see SI Section IV).

**Figure 4:**
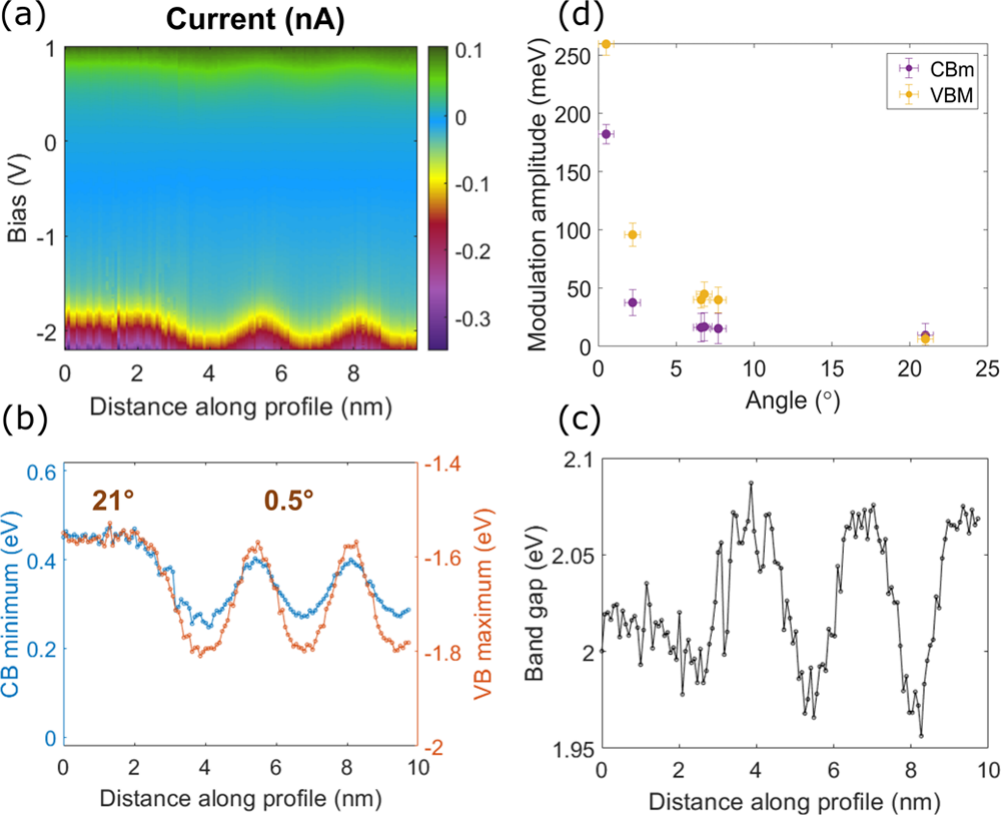
(a) *I*(*V*, *r⃗*) line-cut along the black lines in Figure 3i–l, illustrating the spectroscopic evolution from a 21° to a 0.5° twist angle heterostructure. (b) Plots of the VBM, CBm, and (c) band gap as a function of position along the trace in (a). (d) Modulation amplitude of the CBm and VBM as a function of the twist angle.

The three main results of our experiments can be graphically seen in Figure 3: (i) the local density of states (DOS) is modulated at the moiré pattern wavelength; (ii) the modulation amplitude is significantly larger for the valence band than for the conduction band, and so Δ(*r⃗*) is also modulated at the moiré pattern wavelength; (iii) the modulation amplitude of VBM(*r⃗*) and of CBm(*r⃗*) are decreasing with increasing twist angle. The vanishing spatial modulations of the band edges and of the gap with increasing twist angle are most strikingly seen in Figure 3i–l and in Figure 4. They show a domain wall between a 21° and a 0.5° twist angle region spanned by a single MoS_2_ ML, which completely excludes any origin other than the twist angle for the observed differences. In Figure 4d, we plot the modulation amplitudes of VBM(*r⃗*) and CBm(*r⃗*) (defined as the difference between maximum and minimum band edge energies for each twist angle) as a function of twist angle. The plot clearly shows the monotonic reduction of the VBM(*r⃗*) and CBm(*r⃗*) modulation amplitudes with an increasing twist angle.

Density functional theory (DFT) calculations show that hybridization of MoS_2_ with Au(111) happens primarily through sulfur *p*-orbitals and gold *d*-orbitals, and that it is strongest when the two atoms are positioned atop each other (on-top alignment).^35^ Calculations further show that the hybridization mainly involves out-of-plane orbitals—which define the valence band (VB)—and fewer in-plane orbitals—which define the conduction band (CB).^32^ This is consistent with the greater modulation of the VBM compared to the CBm in Figure 3 and Figure 4a–c. The modulation of the gap at the moiré periodicity in Figure 3d, h, l and in Figure 4c is a direct consequence of the different responses of the CB and of the VB to the hybridization. The effect is strongest in the middle of the bright moiré maxima seen in the topographic images in Figures 1 and 3. These moiré maxima must therefore correspond to the on-top alignment positions in the heterostructures, in agreement with previous assessments.^33,34^

Constant current STM images are a convolution of morphologic and electronic features.^36^ Therefore, it is not a priori clear whether the moiré superstructure observed by STM is a structural modulation or an electronic modulation. To address this question, we examined d*I*/d*V*(*V*, *r⃗*) conductance maps as a function of bias. The fact that the moiré contrast fully inverts for certain bias ranges (SI Section VII) and that the moiré modulation amplitude strongly depends on the imaging bias provides strong evidence that the moiré pattern is primarily of electronic origin in a very flat MoS_2_ ML, in agreement with STM and X-ray standing wave measurement by Silva et al.^34^

Considering a flat MoS_2_ ML on Au(111), we construct a simple model to understand the twist angle dependence of the electronic structure. We assume that the hybridization is primarily determined by the nearest neighbor distances (NND) between the S and Au atoms shown as green and yellow spheres in Figure 5a, respectively. The schematic top views in Figure 5b and c illustrate the changing registry of these two atomic layers as a function of twist angle and the resulting moiré patterns with spatially modulated NND. The distribution of the distances between the nearest S and Au atoms is independent of the twist angle (see inset in Figure 5b and c). In particular, the number of S atoms sitting on top of a Au atom per unit area is the same for all twist angles. Based on the registry between S and Au atoms alone, one would thus not expect any twist angle dependence of the amplitude of the modulated hybridization, in contradiction with the experimental observation. However, looking only at the NND to quantify the hybridization is not physically plausible: it neglects screening which prevents abrupt changes in the charge distribution over very short distances.^37^ To take this into account, we introduce an effective distance *d*_eff_ obtained by convoluting the spatial distribution of NNDs with a Gaussian. By construction, *d*_eff_ is spatially modulated at the moiré period and provides a measure of the strength of the local hybridization: it is stronger where *d*_eff_ is smaller.

**Figure 5:**
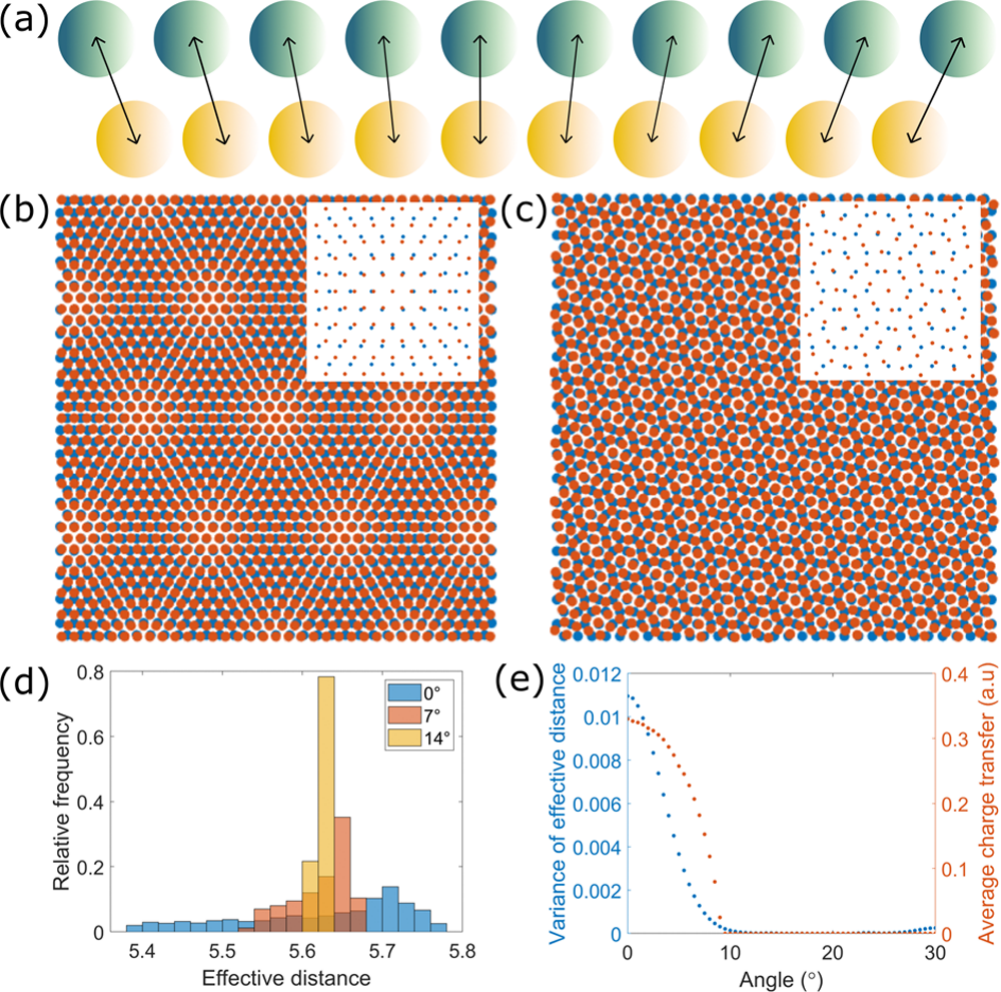
(a) Schematic side view of the bottom sulfur (green) on the top Au (yellow) layers of the heterostructures. Schematic top view of a (b) 0° and a (c) 20° twist angle moiré pattern with Au atoms in blue and S atoms in red. (d) Histogram of the effective distances *d*_eff_ in a 0°, 7° and 14° twist angle heterostructure. (e) Twist angle dependence of the variance of the effective S–Au distance (σ^2^(*d*_eff_), left blue axis) and of the average charge transfer (Δ*Q*, right orange axis).

While the distribution of the distances between nearest S and Au atoms does not depend on twist angle, their spatial distribution does, with significantly larger site-to-site variations at larger twist angles, as seen in Figure 5b and c. These abrupt changes are attenuated by screening, leading to an increasingly narrow distribution of *d*_eff_, as shown by the histograms in Figure 5d for 0°, 7°, and 14° twist angles, which correspond to an increasingly homogeneous *d*_eff_. To assess the amplitude of the modulated hybridization, we plot the variance of *d*_eff_ (which reflects the width of its distribution) as a function of the twist angle in Figure 5e. This clearly shows that the spatial variation of the hybridization vanishes with an increasing twist angle, which explains the correlation between large twist angles and more homogeneous electronic properties observed in Figures 3 and 4.

The average of the VBM and the average of the CBm both shift down in energy, with a larger shift for smaller twist angles (Figure 4b). This indicates stronger electron doping with a larger overall charge transfer when the twist angle is small. The amount of charge transferred at a given site *i* is a function of the local *d*_eff_(*r⃗*_*i*_): Δ*Q*_*i*_ = *f*(*d*_eff_(*r⃗*_*i*_)). The average charge transfer (i.e., charge transfer per Au–S pair for *N* pairs) is
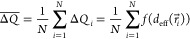
1

To gain insight into the twist angle dependence of Δ*Q* within our simple model, we note that although the distribution of *d*_eff_ depends on the twist angle, its spatial average is constant (SI Section VIII). It means that *f*(*d*_eff_(*r⃗*_*i*_)) is not a linear function. We consider two simple nonlinear *f*(*d*_eff_(*r⃗*_*i*_)) models which reproduce a stronger charge transfer for the Au–S pairs where *d*_eff_ is shorter. Both models yield the same qualitative result.

The first model is motivated by the finding of Silva et al.^34^ in MBE-grown films (0.5° twist angle) that all the bottom S atoms of the MoS_2_ ML fall into either of two categories: strongly bound or weakly bound to the underlying Au atom. Thus, we consider two kinds of S atoms: one that contributes significantly (the strongly bound) and one that does not contribute to the charge transfer (the weakly bound). Assuming a given fraction (e.g., 1/3) of significantly contributing S atoms in the 0° twist angle heterostructure, we can estimate a general cutoff *d*_eff,c_ below (above) which all S–Au pairs fall in the strongly (weakly) bound category with significant (negligible) charge transfer at a given twist angle. In this case
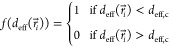
2In Figure 5e (right orange axis) we show Δ*Q* as a function of twist angle calculated using eq 2. We find that Δ*Q* monotonically decreases as a function of the twist angle, which reproduces the observed overall decreasing charge transfer with an increasing twist angle. We obtain the same result with another nonlinear function *f*(*d*_eff_(*r⃗*_*i*_)) = 1/*d*_eff_, as shown in SI Section VIII.

In summary, we present a systematic study of the twist-angle-dependent electronic properties of exfoliated MoS_2_ monolayers on Au(111) using high-resolution scanning tunneling microscopy and spectroscopy. We find that the conduction and valence bands are modulated at the moiré pattern period. The modulations are most prominent in the valence band and largest for the smaller twist angles. They vanish with increasing twist angles. We propose a simple model to understand this twist angle dependence based on a changing hybridization between S and Au orbitals, which depends not only on the relative positions of the nearest S and Au atoms but also on their neighboring configurations. These findings provide detailed insight into designing monolayer-on-metal heterostructures with variable electronic properties and doping by adjusting the twist angle. They also provide a platform to explore correlated and ordered electronic phases in combination with periodic charge transfer (or doping) patterns.

## Methods

### Sample Preparation and Characterization

We use gold-assisted exfoliation^21,23,27^ onto template-stripped gold substrates^24,38^ to mechanically isolate MoS_2_ monolayers (MLs). The gold substrates are prepared in-house. First, we evaporate gold onto a clean ultraflat silicon (Si) wafer. Second, we epoxy another flat Si piece onto the crystalline gold film. We then cleave this sandwich at the evaporated Au–Si interface to get an ultraflat gold surface that reflects the flatness of the original Si substrate. Bulk 2H-MoS_2_ single crystals were obtained from HQ graphene. These crystals are exfoliated to the monolayer limit onto the freshly exposed Au surface in a nitrogen-filled glovebox, using scotch-tape exfoliation. The strong affinity between sulfur and the very clean and flat template-stripped Au substrate allows us to obtain millimeter-sized MoS_2_ MLs. We identify ML MoS_2_ flakes based on their optical contrast on gold and using Raman spectroscopy. A detailed characterization of the gold substrate and of the MoS_2_-on-Au heterostructures is presented in SI Section I and SI Section III. Landing the STM tip on a desired region on the flake is performed using optical microscopy images. Throughout the process, we carefully protect the samples from exposure to the ambient atmosphere to avoid contamination and device degradation. For Raman and optical measurements, the samples were placed in a customized airtight container with optical access that can be sealed in a glovebox. For transferring the samples from the glovebox to the STM, we used a home-built vacuum suitcase which can be directly attached to the load-lock of the ultrahigh vacuum STM chamber. Prior to the STM measurements, the samples were annealed in situ at 150 °C for about 100 h to obtain an optimal surface.

### STM/STS Characterization

All the scanning tunneling microscopy and spectroscopy experiments were done using a Specs JT Tyto STM at 77.7 K (0.5° and 21° twisted heterostructures) or 5 K (all other heterostructures), at a base pressure better than 1 × 10^–10^ mBar. We used electrochemically etched W or Ir tips, all carefully conditioned and characterized in situ on a Au(111) single crystal. STM topographic images were recorded in a constant current mode. d*I*/d*V*(*V*) conductance curves were acquired by using a lock-in with a sample bias modulation amplitude of 15 mV at 527 Hz.

### Modeling

For the qualitative modeling of the twist-angle-dependent hybridization, at each twist angle, we first generate the positions of the S and Au atoms (*R⃗*_*i*_^S/Au^) in triangular lattices lying in parallel planes separated by a distance *d*_0_ (for a side and top view see Figure 5a-c). Then, for each gold atom, we identify the nearest sulfur atom and determine their separation: *d*_NND_(*R⃗*_*i*_^*Au*^). Finally, to obtain *d*_eff_(*r⃗*_*i*_) we spatially convolve the *d*_NND_(*R⃗*_*i*_^*Au*^) field with a Gaussian kernel. For all further calculations (statistical quantities, average charge transfer), we discard a small region near the edges, i.e. we only consider the region where the convolution kernel is entirely within the lattice. At every twist angle, we used a large number of atoms (on the order of 10^6^) in each layer to minimize finite size and edge termination effects.

## Supporting Information Available

Substrate and sample characterization; moiré pattern imaging distortions; twist angle calculation; characterization of gold grains and their interface; nanobubbles; spatial mapping of VBM, CBm, gap using *I*(*V*, *r⃗*) curves; d*I*/d*V*(*V*, *r⃗*) as a function of bias, average charge transfer as a function of twist angle (PDF)
