# Supplementary material for: Twist-Angle-Dependent Electronic Properties of Exfoliated Single Layer MoS2 on Au(111)
Source: Nano Lett. 2023 Oct 16;23(20):9406–12. doi: 10.1021/acs.nanolett.3c02804 (PMC10603799; doi:10.1021/acs.nanolett.3c02804)
Supplement: Supplementary file 1 — nl3c02804_si_001.pdf [file nl3c02804_si_001.pdf]

Supporting Information for Twist-Angle-Dependent Electronic Properties of Exfoliated Single Layer MoS_2 on Au(111)

Ishita Pushkarna,[1,2] Árpád Pásztor,[1,2,*] and Christoph Renner[1,†]

[1] *DQMP, Université de Genève, 24 Quai Ernest Ansermet, CH-1211 Geneva, Switzerland*

[2] *These authors contributed equally to this work.*

CONTENTS

I. Substrate and sample characterization 2

II. Determination of the twist angle 4

III. Characterization of gold grains and their interfaces 5

IV. Moiré pattern deformation due to imaging distortions 7

V. Nanobubbles on the surface 8

VI. Spatial mapping of VBM, CBm, and gap using $I(V, \vec{r})$ curves 9

VII. Conductance maps ($dI/dV(V, \vec{r})$) as a function of bias 10

VIII. Twist-angle-dependent average charge transfer 14

References 14

* arpad.pasztor@unige.ch

† christoph.renner@unige.ch

I. SUBSTRATE AND SAMPLE CHARACTERIZATION

We obtained millimeter-sized monolayer (ML) MoS_2 by exfoliating 2H-MoS_2 single crystals onto template-stripped gold substrates. Exfoliation onto these substrates primarily resulted in large MLs, with only a few tiny thicker flakes. A typical ML is outlined in black in the optical microscope image in Suppl. Fig. 1(a).

Exfoliated MLs were identified through the characteristic Raman spectra of MoS_2 on Au [1, 2]. Due to the strong interaction with the substrate, ML MoS_2 has a markedly different Raman spectrum on gold (Suppl. Fig. 1(b), blue spectrum) than on SiO_2/Si [3]. The A_{1g} and E_{2g} modes in the ML are shifted with respect to their bulk positions on both substrates. However, the shift is different on Au, with an additional splitting of the A_{1g} mode appearing around 397 cm^{-1} (Suppl. Fig. 1(b), blue spectrum). The bilayer spectrum features four distinguishable peaks (Suppl. Fig. 1(b), orange spectrum), which correspond to the combined peaks observed in bulk and ML specimens. This spectrum can be understood as a combined contribution from a bulk spectrum and from the first ML, which is affected by the substrate. The evolution of Raman peaks as a function of MoS_2 flake thickness is summarized in Suppl. Fig. 1(c) where we show the position of the peaks at several different locations on each flake.

The gold substrates were characterized using X-ray diffraction (XRD) and atomic force microscopy (AFM). The XRD spectrum in Suppl. Fig. 1(d) shows a peak around 38.1°, indicating (111) orientation, and a peak near 81.81° corresponding to the (222) reflection of the gold surface. The peaks at 34° and 69.25° correspond to the Si(100) substrate [4, 5]. The large-scale AFM image in Suppl. Fig. 1(e) reveals an ultra-flat gold surface, with a typical roughness of less than a nanometer. Disposing of such flat and freshly exposed gold surfaces is essential to exfoliate large-area MoS_2 MLs. The polycrystalline structure of the gold substrate is nicely resolved in smaller range AFM (Suppl. Fig. 1(f)) and STM images (Suppl. Fig. 1(g)).

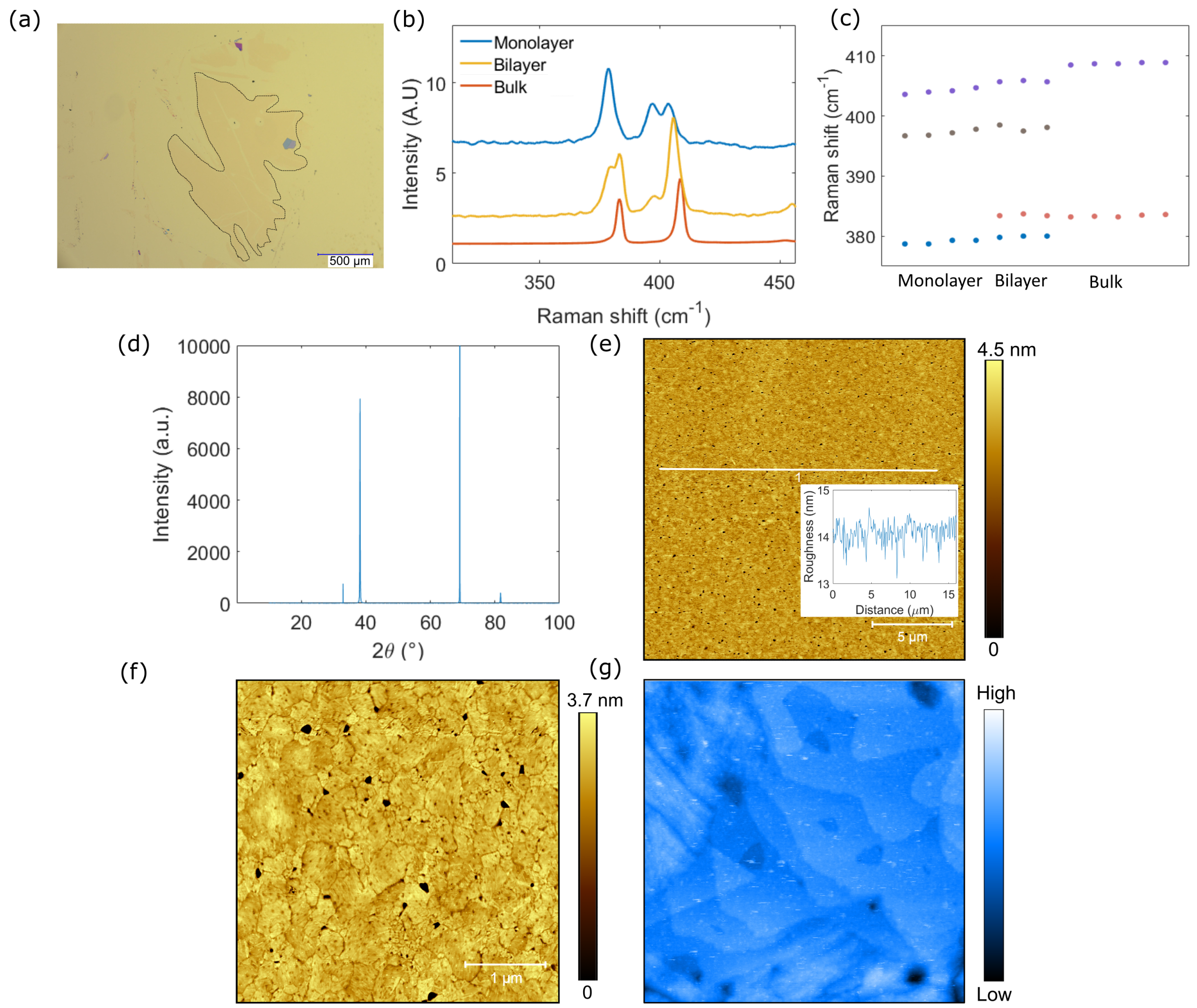

Suppl. Fig. 1. (a) Optical microscope image showing a millimeter-sized monolayer MoS_2 flake (black outline) on the gold substrate. (b) Raman spectra of monolayer (blue), bilayer (yellow), and bulk (orange) MoS_2 on Au. (c) Raman peak positions for three different MoS_2 flake thicknesses, with data taken at different positions on each flake. (d) XRD spectrum of the bare Au substrate, showing its (111) orientation (38.1° and 81.81° peaks). (e) 20×20 μm^2 AFM image of the gold substrate, illustrating the ultra-flat surface over tens of microns. (f) 4×4 μm^2 AFM image showing the polycrystalline nature of the substrate. (g) 0.2×0.2 μm^2 STM image of the same substrate revealing gold terraces.

II. DETERMINATION OF THE TWIST ANGLE

We determine the twist angle between the MoS_2 lattice and the Au(111) surface based on the Fourier-transform (FT) of atomically resolved STM topographies exemplified in Suppl. Fig. 2(a). We identify and select the peaks corresponding to the moiré and to the MoS_2 wave vectors marked by green and red circles in Suppl. Fig. 2(b), respectively, and calculate the length of each wave vector. The length of the gold lattice wave vector is given by the sum of the wave vectors of MoS_2 and moiré lattices. To determine the gold lattice wave vector we calculate all the possible sums and choose the pairs of moiré and MoS_2 wave vectors which give the closest value to the lattice constant of gold. Then, knowing $\vec{k}_{\mathrm{MoS_2}}$ and $\vec{k}_{\mathrm{Au}}$, we calculate the twist angle between the MoS_2 and gold lattices using supplementary equation (1). In the example of Suppl. Fig. 2, we find a twist angle of 7.7°. The uncertainty on the twist angles determined in this way is ±0.5°, primarily limited by the precision of measuring the k-vectors in the FTs.

$$cos(\varphi) = \frac{\vec{k}_{\mathrm{Au}} \cdot \vec{k}_{\mathrm{MoS_2}}}{|\vec{k}_{\mathrm{Au}}||\vec{k}_{\mathrm{MoS_2}}|} \qquad (1)$$

An alternative approach to determine the twist angle is to calculate the moiré wavelength as a function of the twist angle. This allows to extract the twist angle from the plot of $|\vec{k}_{\mathrm{Moiré}}|/|\vec{k}_{\mathrm{MoS_2}}|$ as a function of twist angle (Suppl. Fig. 2(c)).

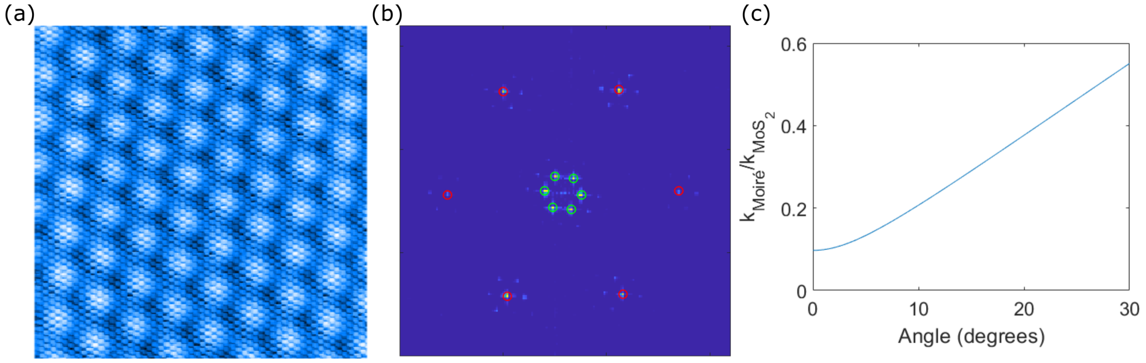

Suppl. Fig. 2. (a) 15×15 nm^2 STM topography of a MoS_2 ML on Au(111) aquired at 300 mV and 100 pA set point. (b) Corresponding FT with the MoS_2 lattice (red) and moiré (green) peaks selected to determine the twist angle (7.7° in this case). (c) Plot of $|\vec{k}_{\mathrm{Moiré}}|/|\vec{k}_{\mathrm{MoS_2}}|$ as a function of twist angle.

III. CHARACTERIZATION OF GOLD GRAINS AND THEIR INTERFACES

XRD shows that the gold film is composed of [111]-oriented grains. High-resolution STM topography is consistent with XRD, and provides atomic-scale insight into the tilting and rotation of the grains about their [111]-axis. In Suppl. Fig. 3, we analyze a high-resolution topographic image of a continuous MoS_2 ML spanning two adjacent gold grains. We can determine the step heights in each grain by successively flattening the STM image with respect to a terrace on the left-hand side grain and then with respect to a terrace on the right-hand side grain. In both cases, we extract a height difference between the terraces of about 228 pm, consistent with the step height expected for Au(111) [6, 7], as shown in the lower panels of Suppl. Fig. 3(a),(b).

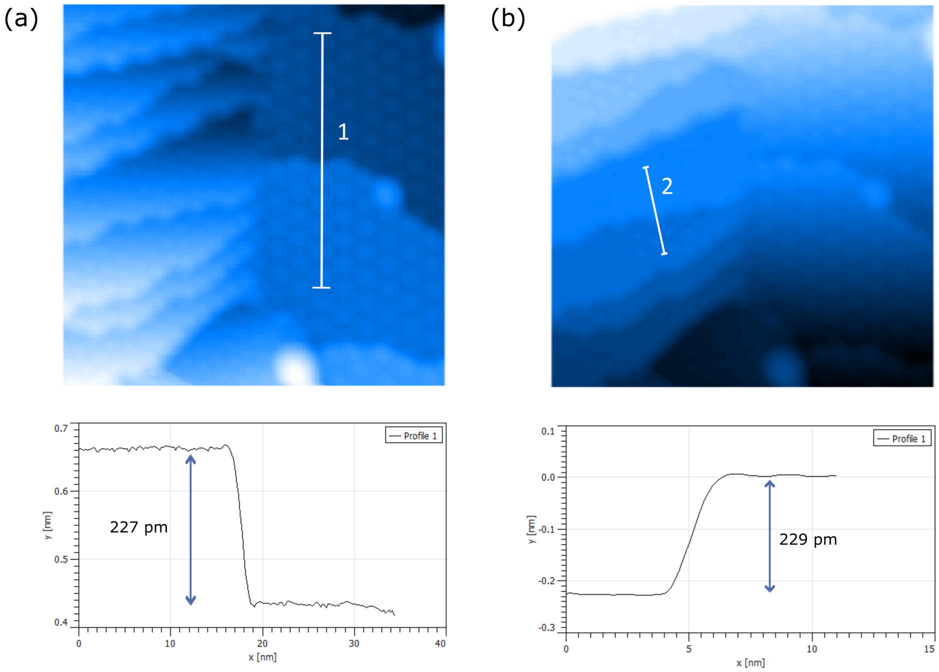

Suppl. Fig. 3. 50×50 nm^2 topographic STM image of two adjacent Au(111) grains leveled to show the terraces in the right-hand grain in (a) and the left-hand grain in (b), with corresponding height profile along the white line in each background corrected image.

The same topographic STM image as in Suppl. Fig. 3 is shown in Suppl. Fig. 4(a), but leveled to highlight the moiré patterns in the two adjacent grains. A closer look at the grain boundary region in Suppl. Fig. 4(b) clearly shows a continuous MoS_2 flake extending over

the entire field of view. The changing moiré pattern is thus a direct consequence of the different orientations of the two Au(111) grains. Continuous MoS_2 flakes spanning Au(111) grain boundaries provide a unique platform to study the electronic properties as a function of twist angle in a single device with the same tip, excluding any spurious experimental effects that might affect the data.

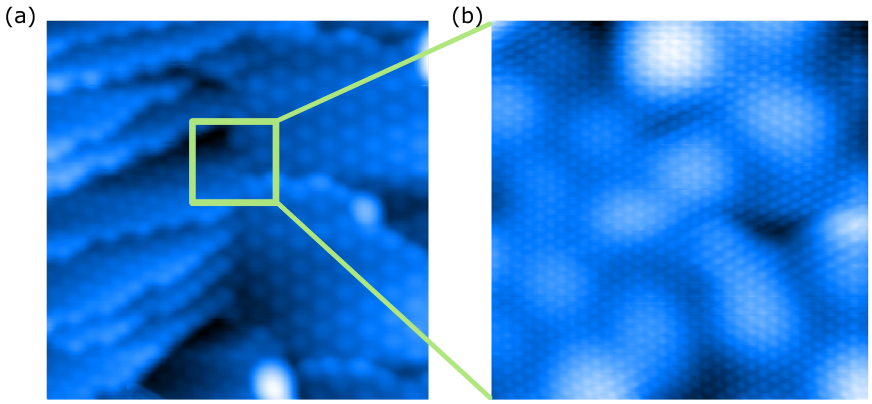

Suppl. Fig. 4. (a) 50×50 nm^2 high resolution STM image (same as in Suppl. Fig. 3) levelled to highlight the moiré patterns. (b) Magnified 10×10 nm^2 grain boundary region to emphasize the continuous MoS_2 lattice. The large balls correspond to the moiré pattern and the small ones to the MoS_2 atomic lattice.

IV. MOIRÉ PATTERN DEFORMATION DUE TO IMAGING DISTORTIONS

Some moiré patterns appear distorted, e.g. in Fig. 1(a) and Figs. 3(i)-(l). These deformations are due to imaging distortions (piezo creep and thermal drift) which are naturally present in images that take a long time to acquire, especially at the higher experimental temperatures as in the case of Fig. 1(a) and Figs. 3(i)-(l). We illustrate this in Suppl. Fig. 5, where we show the topographic image taken during the lengthy acquisition of the conductance map in Suppl. Fig. 5(a) (same as the data presented in Fig. 3), and a fast high-resolution topographic image of the same region taken just before mapping the conductance in Suppl. Fig. 5(b) atop their respective FT. The nearly perfect hexagonal lattice and moiré pattern in Suppl. Fig. 5(b), which took much less time to record, confirms that the distortion in Suppl. Fig. 5(a) is an imaging artifact.

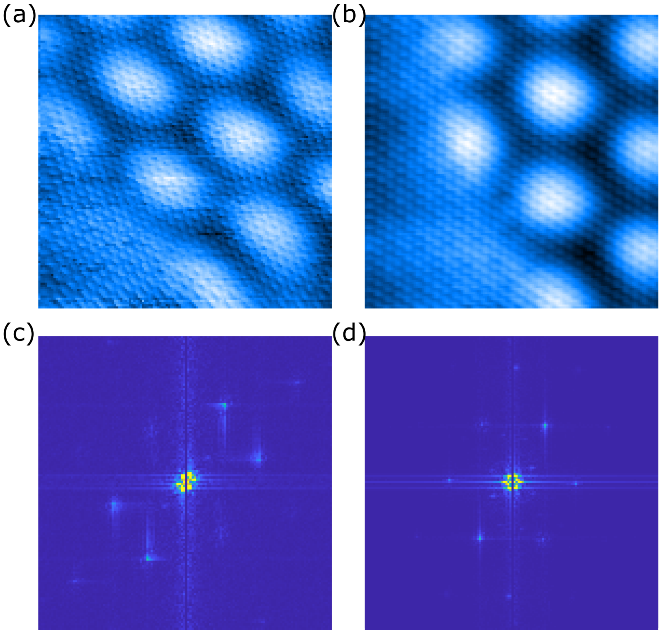

Suppl. Fig. 5. Long and short acquisition time topographic images of the same region to illustrate the effect of drift. (a) Same image as in Fig. 3(i) of the main text acquired simultaneously with a dense $I(V, \vec{r})$ map and (c) corresponding FT. The distortion amounts to about 37%. (b) High-resolution topographic image of the same region acquired just before the image in panel (a) and much more rapidly without the spectroscopic mapping, and (d) corresponding FT with hardly any distortion visible. Both images were only leveled by subtracting a linear fit from each line without any further treatment.

V. NANOBUBBLES ON THE SURFACE

The large area topographic STM image in Suppl. Fig. 6(a) shows a single continuous MoS_2 ML extending over the entire image and spanning different Au(111) grains and terraces. We observe occasional bubbles, usually located around step edges (Suppl. Fig. 6(b)). They correspond to regions where the MoS_2 ML is decoupled from the substrate. Similar features have been observed in other studies of exfoliated TMDs [8] and graphene [9] on different substrates. These decoupled regions show purely semi-conducting $I(V)$ spectra (Suppl. Fig. 6(c), orange spectrum), whereas the surrounding regions show hybridized spectra, with a reminiscence of the semi-conducting nature of MoS_2 (Suppl. Fig. 6(c), blue spectrum).

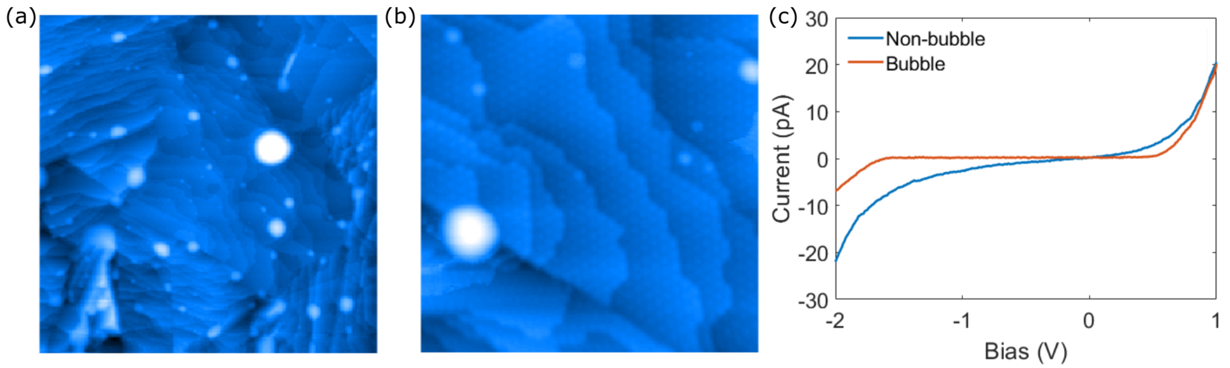

Suppl. Fig. 6. (a) 400×400 nm^2 STM topography of a single continuous MoS_2 ML spanning different Au(111) grains and terraces. (b) Magnified 100×100 nm^2 STM topography revealing the MoS_2 lattice and moiré patterns on every gold terrace where MoS_2 is in close contact with the Au(111) surface, and nanobubbles where MoS_2 is detached from the gold substrate. (c) Semi-conducting $I(V)$ spectrum measured on a bubble (orange), and a hybridized $I(V)$ spectrum measured off the bubble (blue).

VI. SPATIAL MAPPING OF VBM, CBM, AND GAP USING $I(V, \vec{r})$ CURVES

Here we show how we extract the CB and VB edges from $I(V)$ spectra by fitting the $I(V)$ curves following Zhuou et al. [10] with a slightly different model DOS. We considered a constant DOS for the purely semi-conducting decoupled MoS_2 areas. However, for the hybridized films, a better fit is obtained using a square-root energy-dependent semiconducting DOS (3D) in each band. To take into account the metallic background due to the hybridization, we added a non-zero constant to the DOS over the entire energy range. The VB and CB regions were then fitted separately, using independent constants for each half of the spectrum (each spectrum is divided into two parts at the approximate center of the gap near -0.5 V). This ensures a roughly equal number of data points to fit both sides. In Suppl. Fig. 7(a), we show an example of a fitted $I(V)$ spectrum.

Suppl. Fig. 7(b), (c), and (d) show the spatial mapping of VBM, CBm, and the band gap, obtained by fitting $I(V)$ curves. Both methods (fitting of $dI/dV(V)$ or $I(V)$ spectra) give the same information on band edge modulation and attest the suitability of any of the two methods.

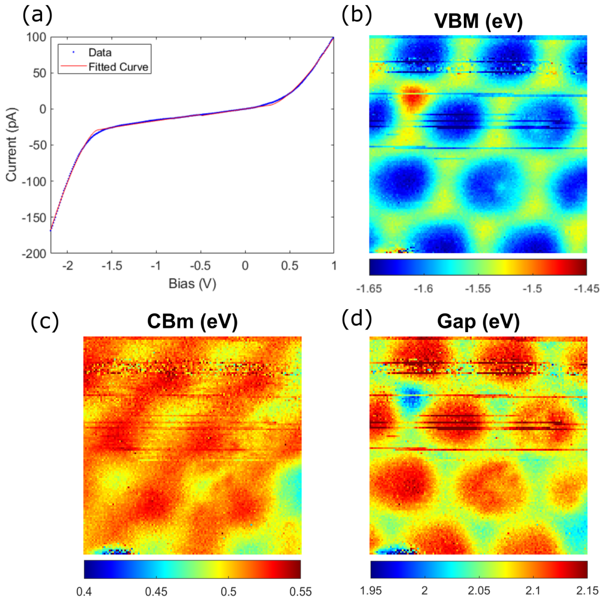

Suppl. Fig. 7. (a) $I(V)$ spectrum and corresponding fit. (b) VBM, (c) CBm, and (d) gap modulation of the 2.2° moiré pattern extracted from a 8×8 nm^2 I(V,$\vec{r}$) map acquired in the same area as Fig. 3(a).

VII. CONDUCTANCE MAPS ($dI/dV(V,\vec{r})$) AS A FUNCTION OF BIAS

To address the electronic or structural origin of the moiré pattern observed in STM images, we examined the $dI/dV(V,\vec{r})$ conductance maps of a 2.2° twist angle heterostructure as a function of bias in Suppl. Fig. 8. The main observations are the following: i) we do not see any significant modulation of the local density of states (LDOS) at bias voltages inside the MoS_2 gap (Suppl. Fig. 8(c)), probably because the tip is stabilized far outside the gap and the signal from these low energy states is too weak; ii) outside of the gap, the LDOS is modulated at the moiré period (Suppl. Fig. 8(a),(b),(d),(e)); iii) the moiré contrast inverts at least three times in the examined energy range, once at negative bias (Suppl. Fig. 8(a)-(b)), once across the gap (Suppl. Fig. 8(b)-(d)), and once at positive bias (Suppl. Fig. 8(d)-(e)). Such contrast inversions in the conductance maps as a function of energy are not consistent with pure morphologic features: hills cannot swap their positions with trenches three times as a function of imaging bias.

The predominantly electronic origin of the observed moiré pattern can be appreciated by comparing the $dI/dV(V)$ curves measured at a peak position and at a valley position: they cross each other at several biases and are nearly equal in the gap region (see Suppl. Fig. 10(a) and Fig. 2(b)). Therefore, depending on the bias chosen to extract the conductance maps, the LDOS will be largest when the tip is over a crest or when it is over a valley of the moiré, leading to changing and inverted contrasts. To make sure the bias set point does not affect these observations, we did the same analysis for a $dI/dV(V,\vec{r})$ map acquired at a negative bias set point of -3 V. The result is exactly the same as seen in Suppl. Fig. 9.

The changing contrast of the moiré pattern in Suppl. Fig. 8 and Suppl. Fig. 9 is a direct consequence of the different tunneling spectra measured when the STM tip sits at a crest or at a valley of the moiré pattern (Suppl. Fig. 10(a)). The moiré pattern contrast can be quantified by summing the intensity of the six peaks corresponding to the moiré pattern in the Fourier transforms (FTs) of the conductance maps extracted from $dI/dV(V,\vec{r})$. This total intensity is plotted as a function of bias voltage in Suppl. Fig. 8(f) and Suppl. Fig. 9(f). The color code is defined in the following way: red when the conductance is larger at the moiré crest, corresponding to the contrast in Suppl. Fig. 8(a),(d); green when the conductance is larger in the moiré valley, corresponding to the contrast in Suppl. Fig. 8(b),(e); blue when the conductances are nearly the same at the moiré crest and in the moiré valley, correspond-

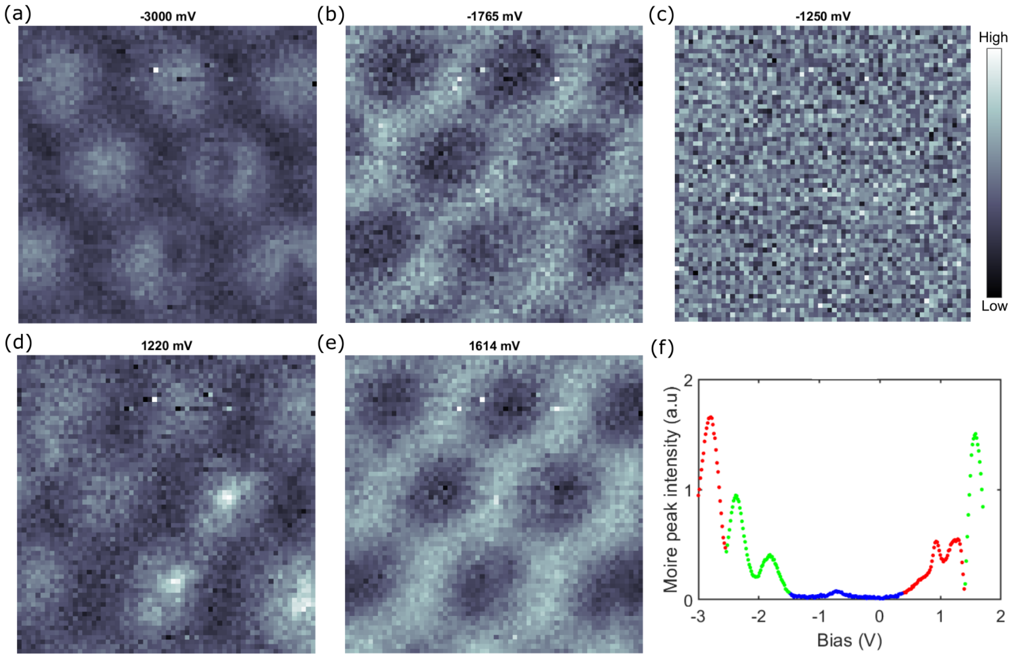

Suppl. Fig. 8. 8×8 nm^2 conductance maps extracted from a $dI/dV(V, \vec{r})$ map of a 2.2° twist angle heterostructure at (a) -3000 mV, (b) -1765 mV, (c) -1250 mV, (d) 1220 mV, and (e) 1614 mV. Set point during the conductance map: I_t= 100 pA; V_b= 1.7 V. (f) Plot of the total intensity of the six moiré peaks in the FT of all conductance maps available between -3 V and 1.7 V. Red corresponds to the contrast in panel (a), green to the contrast in panel (b), and blue to energies where the moiré peaks are not resolved in the FT.

ing to the contrast in Suppl. Fig. 8(c). This bias dependence of the moiré contrast can be directly compared to the numerical difference between the two conductance curves measured at the moiré crest and moiré valley positions in Suppl. Fig. 10(a). In Suppl. Fig. 10(c), we plot $dI/dV(V, \text{crest}) - dI/dV(V, \text{valley})$, with a positive (negative) result represented by a red (green) dot. This curve is remarkably similar to the one extracted from the Fourier analysis of the conductance maps and confirms the predominantly electronic origin of the moiré pattern contrast.

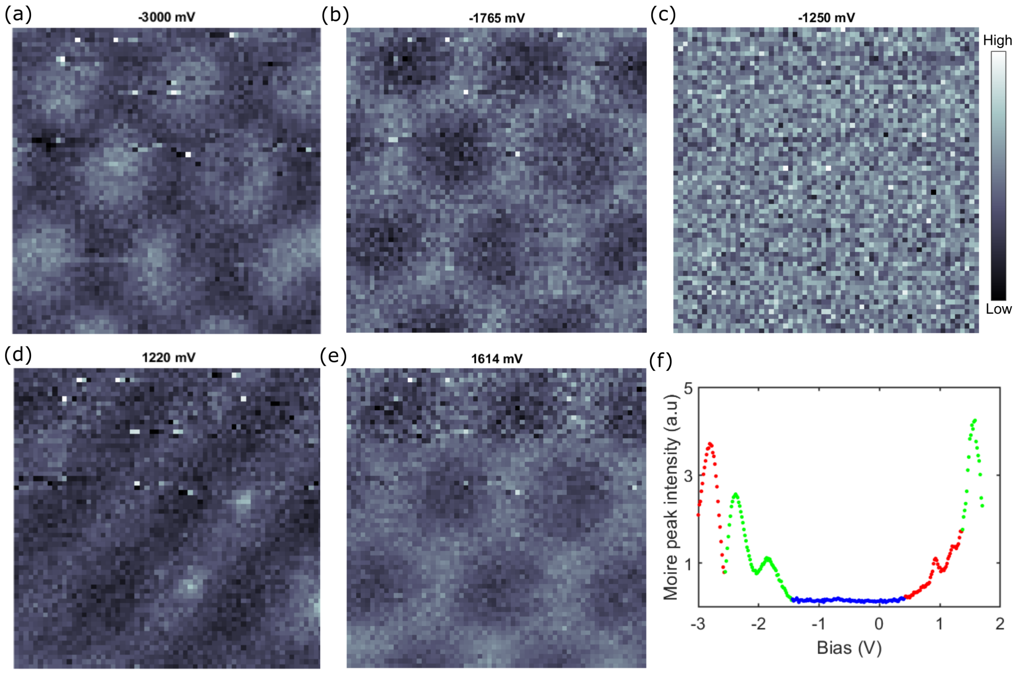

Suppl. Fig. 9. 8×8 nm^2 conductance maps extracted from a $dI/dV(V,\vec{r})$ map of a 2.2° twist angle heterostructure at (a) -3000 mV, (b) -1765 mV, (c) -1250 mV, (d) 1220 mV, and (e) 1614 mV. Set point during the conductance map: I_t= 100 pA; V_b= -3 V. (f) Plot of the total intensity of the six moiré peaks in the FT of all conductance maps available between -3 V and 1.7 V. Red corresponds to the contrast in panel (a), green to the contrast in panel (b), and blue to energies where the moiré peaks are not resolved in the FT.

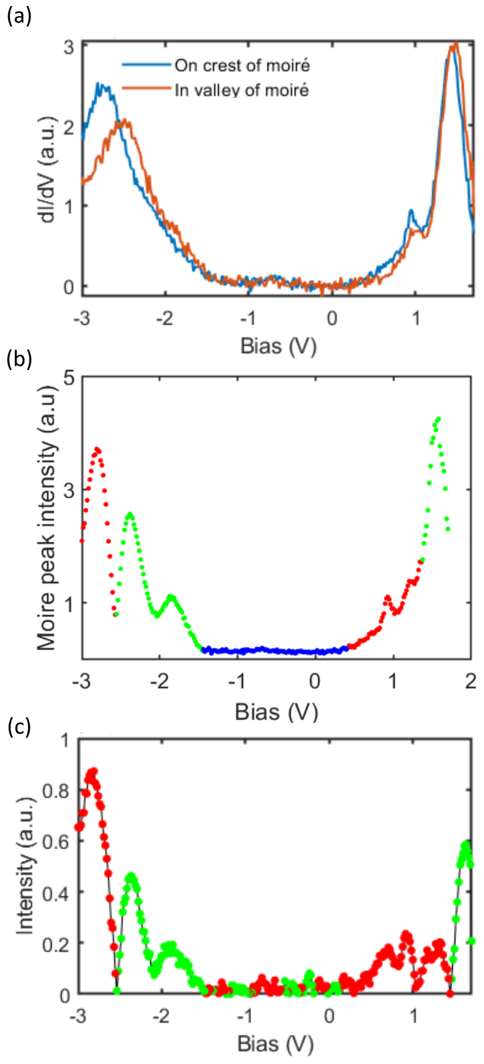

Suppl. Fig. 10. (a) $dI/dV(V)$ spectra measured at the crest (blue) and valley (orange) positions of the moiré pattern. (b) Moiré modulation amplitude extracted from the total intensity of the corresponding six peaks in the FT of the energy-dependent conductance maps measured on a 2.2° twist angle heterostructure (same data as Suppl. Fig. 9(f)). (c) The absolute value of the *on-crest* minus *in-valley* $dI/dV(V)$ spectra in panel (a), where red (green) corresponds to a positive (negative) difference.

VIII. TWIST-ANGLE-DEPENDENT AVERAGE CHARGE TRANSFER

In this model, we consider the charge transfer at a given Au-S nearest neighbor pair to be proportional to $1/d_{\text{eff}}$. We calculate the average charge transfer as:

$$\overline{\Delta Q} = \frac{1}{N}\sum_{i=1}^{N}\frac{1}{d_{\text{eff}}(\vec{r}_i)}. \tag{2}$$

While the average of $d_{\text{eff}}(\vec{r}_i)$ does not change as a function of twist angle (Suppl. Fig. 11(a)) we clearly see in Suppl. Fig. 11(b) –similarly to the model presented in the main text– that the average charge transfer is decreasing with increasing twist angle.

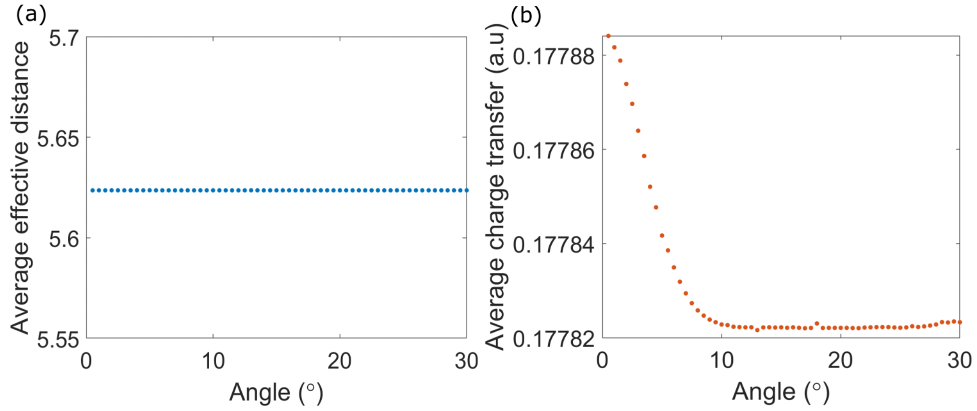

Suppl. Fig. 11. (a) Average of $d_{\text{eff}}(\vec{r}_i)$ and (b) $\overline{\Delta Q}$ in the $1/d_{\text{eff}}$ model as a function of twist angle.

REFERENCES

[1] M. Velický, A. Rodriguez, M. Bousa, A. V. Krayev, M. Vondrácek, J. Honolka, M. Ahmadi, G. E. Donnelly, F. Huang, H. D. Abruña, K. S. Novoselov, and O. Frank, “Strain and charge doping fingerprints of the strong interaction between monolayer MoS_2 and gold,” *The Journal of Physical Chemistry Letters*, vol. 11, no. 15, pp. 6112–6118, 2020.

[2] E. Pollmann, S. Sleziona, T. Foller, U. Hagemann, C. Gorynski, O. Petri, L. Madauß, L. Breuer, and M. Schleberger, “Large-area, two-dimensional MoS_2 exfoliated on gold: Direct experimental access to the metal–semiconductor interface,” *ACS Omega*, vol. 6, no. 24, pp. 15929–15939, 2021.

[3] H. Li, Q. Zhang, C. C. R. Yap, B. K. Tay, T. H. T. Edwin, A. Olivier, and D. Baillargeat, "From bulk to monolayer MoS_2: Evolution of raman scattering," *Advanced Functional Materials*, vol. 22, no. 7, pp. 1385–1390, 2012.

[4] S. Krishnamurthy, A. Esterle, N. C. Sharma, and S. V. Sahi, "Yucca-derived synthesis of gold nanomaterial and their catalytic potential," *Nanoscale Research Letters*, vol. 9, no. 1, p. 627, 2014.

[5] Q. Zhao, J. Xu, X. Y. Xu, Z. Wang, and D. P. Yu, "Field emission from AlN nanoneedle arrays," *Applied Physics Letters*, vol. 85, no. 22, pp. 5331–5333, 2004.

[6] J. V. Barth, H. Brune, G. Ertl, and R. J. Behm, "Scanning tunneling microscopy observations on the reconstructed Au(111) surface: Atomic structure, long-range superstructure, rotational domains, and surface defects," *Physical Review B*, vol. 42, no. 15, pp. 9307–9318, 1990.

[7] X. Sun, M. P. Felicissimo, P. Rudolf, and F. Silly, "NaCl multi-layer islands grown on Au(111)-($22\times\sqrt{3}$) probed by scanning tunneling microscopy," *Nanotechnology*, vol. 19, no. 49, p. 495307, 2008.

[8] J. Pető, G. Dobrik, G. Kukucska, P. Vancsó, A. A. Koós, J. Koltai, P. Nemes-Incze, C. Hwang, and L. Tapasztó, "Moderate strain induced indirect bandgap and conduction electrons in MoS_2 single layers," *npj 2D Materials and Applications*, vol. 3, no. 1, p. 39, 2019.

[9] E. Khestanova, F. Guinea, L. Fumagalli, A. K. Geim, and I. V. Grigorieva, "Universal shape and pressure inside bubbles appearing in van der Waals heterostructures," *Nature Communications*, vol. 7, no. 1, p. 12587, 2016.

[10] X. Zhou, K. Kang, S. Xie, A. Dadgar, N. R. Monahan, X. Y. Zhu, J. Park, and A. N. Pasupathy, "Atomic-scale spectroscopy of gated monolayer MoS_2," *Nano Letters*, vol. 16, no. 5, pp. 3148–3154, 2016.
